# Which factors help to determine the long-term response to first-line tyrosine kinase inhibitors in patients with metastatic renal cell carcinoma: A Turkish multicenter study

**DOI:** 10.17305/bb.2024.10512

**Published:** 2024-12-01

**Authors:** Nargiz Majidova, Mustafa Seyyar, Demet Işık Bayraktar, Gülhan Dinç, Elfag İsgandarov, Javid Huseynov, Alper Yaşar, Abdussamet Çelebi, Nadiye Sever, Erkam Kocaaslan, Pınar Erel, Yeşim Ağyol, Ali Kaan Güren, Rukiye Arıkan, Selver Işık, Özlem Ercelep, Güzin Demirağ, Umut Kefeli, Osman Köstek, İbrahim Vedat Bayoğlu, Murat Sarı

**Affiliations:** 1 Department of Internal Medicine, Division of Medical Oncology, Marmara University School of Medicine, Istanbul, Turkey; 2 Department of Internal Medicine, Division of Medical Oncology, Kocaeli University, Kocaeli, Turkey; 3 Department of Internal Medicine, Division of Medical Oncology, Ondokuz Mayıs University Faculty of Medicine, Samsun, Turkey; 4 Department of Internal Medicine, Division of Medical Oncology, Professor Dr Cemil Tascioglu City Hospital, Istanbul, Turkey

**Keywords:** IMDC score, renal cell carcinoma, tyrosine kinase inhibitor, long-lasting response

## Abstract

In patients with metastatic renal cell carcinoma (mRCC), although immune checkpoint inhibitor (ICI)–tyrosine kinase inhibitor (TKI) combinations or ICI–ICI combinations are typically recommended as first-line treatments, access to these combinations is often limited in developing countries. Therefore, there is a need for predictive markers to identify patients who may achieve long-term responses with single-agent TKIs. Our study aimed to identify such predictive parameters. This multicenter, retrospective study included patients diagnosed with mRCC who received first-line treatment with sunitinib or pazopanib. Patients who did not experience disease progression for 36 months or longer were classified as long-term responders. We investigated the clinical and pathological characteristics predictive of long-term response in these patients. A total of 320 patients from four hospitals were included, with a median age of 60 years (range of 20–89 years). According to the International Metastatic Renal Cell Carcinoma Database Consortium (IMDC) risk classification, 109 patients were in the favorable risk group and 211 in the intermediate-poor risk group. The median progression-free survival (PFS) and overall survival (OS) for all patients were 12.5 months and 76.4 months, respectively. In the long-term responders’ group, the median PFS was 78.4 months. For the entire group, prior nephrectomy, an Eastern Cooperative Oncology Group (ECOG) performance status (PS) <1, and the absence of brain metastasis were predictive factors for long-term response. In the favorable risk group, the absence of brain metastases predicted long-term response. In the intermediate-poor risk group, prior nephrectomy and an ECOG PS <1 was predictive of long-term response. Thus, in certain individuals with mRCC, TKIs can provide a long-lasting response, which can be predicted by nephrectomy, an ECOG PS <1, and the absence of brain metastases.

## Introduction

More than 90% of all cases of kidney cancer are clear cell renal cell carcinoma (RCC), being the most prevalent histological subtype [1]. Nephrectomy is the main medical option for treating local disease. Standard chemotherapy is not effective against metastatic disease, unlike other malignancies. As a result, new treatment options have been developed by looking into the biochemical and morphological traits of this particular cancer type. Tyrosine kinase inhibitors (TKIs), such as sunitinib, pazopanib, axitinib, and cabozantinib, and immunocheckpoint inhibitors, such as nivolumab, pembrolizumab, and avelumab, both as monotherapy and in combination therapy, have emerged as new therapeutic options for metastatic-RCC (mRCC) [2–6].

Based on data from the studies, ICI–TKI combinations appeared to provide better progression-free survival (PFS) and overall survival (OS) as first-line systemic therapies in mRCC patients. In addition, the combination of nivolumab plus ipilimumab (ICI–ICI) appeared to provide higher PFS and OS among patients with high PD-L1 expression. Moreover, the highest complete response (CR) rate was also associated with nivolumab plus ipilimumab [3, 5, 7]. At the same time, we know that there is no OS benefit with combination therapies in patients in the favorable risk group, while some patients in the intermediate-poor risk group can be effectively managed with single-agent treatments.

In a study comparing pazopanib with sunitinib, response rates in mRCC were reported to be 31% vs 25%, median PFS was 8.4 vs 9.5 months, and median OS was 28.4 vs 29.3 months [8, 9]. Drug tolerance is difficult in patients with mRCC due to immune-related side effects associated with immunotherapies, and this situation becomes even more difficult when TKI or other IO are added. In studies, grade 3 side effects and drug discontinuation rates are high in combined therapies [10].

In many developing countries, combination therapy is not economically available. Therefore, it is important for the economies of developing countries to identify predictive factors for patients who achieve long-term response with single-agent TKIs and to use single-agent TKIs in these patients. There is a search in the literature on this subject and Catalano et al. showed that patients with previous nephrectomy, Eastern Cooperative Oncology Group (ECOG) PS < 1, and lack of liver metastasis factors achieved long-term response with single-agent TKIs. In another study, Park et al. showed that favorable responses were achieved with single-agent pazopanib in patients with ECOG PS 0 and previous nephrectomy [11, 12].

In our study, we aimed to determine which patients could achieve long-term treatment response with single-agent pazopanib or sunitinib. By doing so, we aimed to identify the patient subgroup, especially in the favorable risk category, where single-agent TKI may not be sufficient, and the patient subgroup in the intermediate-poor risk category where effective response can be achieved with single-agent TKIs.

## Materials and methods

### Study subjects

**Figure S1.**
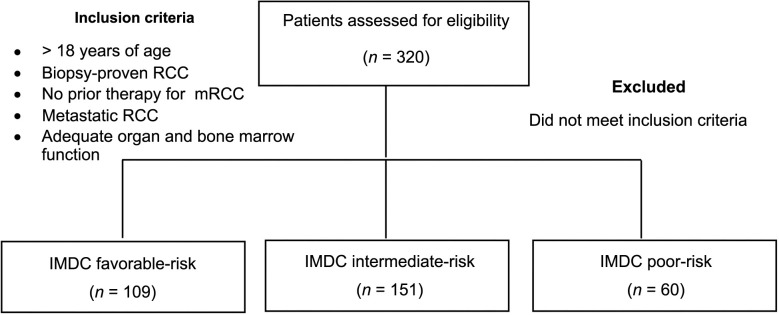
**Flowchart diagram showing the summary of the study design.** RCC: Renal cell carcinoma; IMDC: International Metastatic Renal Cell Carcinoma Database Consortium.

This study is a retrospective multicenter (four centers) analysis of 320 mRCC patients who received sunitinib or pazopanib in first line for mRCC treated between 2008 and 2022 (see Figure S1). Long-term responders were those whose PFS lasted longer than 36 months. Patients were divided into two groups based on their responses over a period of 36 months: short term and long term. All patients’ clinical and demographic details were assessed.

### Ethical statement

This study was performed in line with the principles of the Declaration of Helsinki. Approval was granted by the Ethics Committee of the University of Marmara (Approval Number 02.09.2022.1115).

### Statistical analysis

Treatment responses of all patients were evaluated with imaging methods accepted as standard in their own centers. Prognostic analysis was calculated based on OS (defined as the time between the diagnosis of metastatic disease and date of last known alive or death) and PFS (defined as the time from the first day of first-line TKIs to the date of disease progression or death). Data analysis was performed using SPSS 22.0. Continuous variables were expressed as a median (interquartile range) while categorical variables were expressed as a number (*n*) and percentage (%). Categorical measurements were analyzed using a chi-square test. The Kaplan–Meier method was used to estimate the mean–median OS and DFS rates. The log-rank test was used to compare survival distributions between groups. Logistic regression analysis was used to assess the factors influencing long-term PFS during TKI treatment. Multivariate analysis was calculated using the Cox regression method. A *P* value of <0.05 was considered significant for all tests.

RECIST (Response Evaluation Criteria In Solid Tumors) was used to measure treatment responses. CR as disappearance of all lesions, partial response (PR) was defined as a disease reduction of more than 30% and no new or progressed lesion, progressive disease (PD) was characterized as one that produced additional lesions or a tumor that grew by more than 20% of its initial size, and stable disease as no PR – no PD 50% [5].

## Results

### Characteristics of patients according to treatment response status

Retrospective evaluation of 320 patients was done in our study. Fifty-six patients (17.5%) who received first-line TKI therapy had PFS of 36 months or longer and and these patients were considered as the long-term responders. Characteristic features of short-term and long-term responders are summarized in (Table 1).

**Table 1:** Baseline characteristics of patients according to TKI response

	**All** ***n*** **═** **320**	**PFS** **< 36** **months** ***n*** **═** **264** **(82.5%)** **(short-term responder)**	**PFS** **≥ 36** **months** ***n*** **═** **56** **(17.5%)** **(long-term responder)**	***P*** **value**
Age median (range)	60 (20−89)	60 (20−89)	58 (31−81)	0.45
Gender, *n* (%) male	238 (74.3)	198 (75.0)	40 (71.4)	0.53
Histology, *n* (%) clear cell RCC	265 (82.8)	214 (81.0)	51 (91.0)	0.52
Previous nephrectomy, Yes *n* (%)	254 (79.3)	200 (75.7)	54 (96.4)	***0.001***
ECOG PS, *n* (*%*) ≥ 1	142 (44.3)	124 (46.9)	18 (32.1)	***0.02***
Sarcomatoid feature, Yes *n* (%)	46 (14.3)	37 (10.1)	9 (16.0)	0.41
IMDC score, *n* (%) intermediate-poor	211 (65.9)	184 (69.6)	27 (48.2)	***0.002***
*Metastatic sites, n *(*%*)				
Lung	196 (61.2)	162 (61.3)	34 (60.7)	0.92
Liver	56 (17.5)	51 (19.3)	5 (8.9)	0.06
Nodal	145 (45.3)	122 (46.2)	23 (41.0)	0.48
Bone	129 (40.3)	115 (43.5)	14 (25.0)	***0.01***
Brain	41 (12.8)	40 (15.1)	1 (1.7)	***0.007***
*First-line therapy, n (%)*				
Sunitinib	231 (72.1)	192 (72.7)	39 (69.6)	0.64
Pazopanib	89 (27.8)	72 (27.2)	17 (30.3)	
Line of therapy after TKI, *n* (*%*) > 1	176 (55)	158 (59.8)	18 (32.1)	**<** **0** **.** **0** **0** **1**

IMDC: International Metastatic Renal Cell Carcinoma Database Consortium; ECOG PS: Eastern Cooperative Oncology Group performance status scale; PFS: Progression-free survival; RCC: Renal cell carcinoma; TKI: Tyrosine kinase inhibitor.

Median age, gender, and histological type were similar in both groups. Clear cell carcinoma was the most common subtype and was seen in 82.8% of patients. Previous nephrectomy was performed in 79.3% of all patients and was statistically higher in the long-term responder group (*P* ═ 0.001).When short-term responders were compared with long-term TKI responders, the International Metastatic Renal Cell Carcinoma Database Consortium (IMDC) intermediate-poor risk patient percentage (69.6% vs 48.2%; *P* ═ 0.002), and the rate of bone and/or brain metastases was higher (*P* ═ 0.01 and *P* ═ 0.007, respectively). In short-term responders, the rate of patients with an ECOG PS ≥ 1 was higher (*P* ═ 0.02).

We also looked at Table 1 from a different perspective, namely, how many patients with lung, liver, bone, or LN metastasis have a favorable prognosis. With that perspective, 82.6% of lung metastasis, 91% of liver metastasis, 84.1% of nodes, 89.1% of bones, and 97.6% with brain metastasis had poor prognosis. In this way, we see that among others, even liver metastasis is a poor prognostic factor (*P* ═ 0.06), being close to statistical significance. In other words, while PFS was statistically significantly lower than 36 months in patients with bone and brain metastases, it was also clinically significant in patients with liver metastases.

### Clinical feauters of individuals based on IMDC risk score

According to the IMDC risk score, 109 individuals were in the favorable category (see Table S1). Among these individuals, sunitinib was administered to 78 (71.5%) and pazopanib to 31 (28.4%). In the group with long-term response, 29 patients (26.6%) were present. Brain metastases were statistically more common in patients with short-term TKI responders than long-term responders (18.7% vs 3.4% *P* ═ 0.04). These two groups shared similar clinical characteristics that were not statistically significant.

Two hundred eleven patients were in the intermediate-poor risk group according to the IMDC risk score (see Table S2). Only 12.8% of the participants in this subgroup had PFS longer than 36 months. Except for history of nephrectomy, ECOG PS, absence of brain metastasis, and receiving treatment more than one line after TKI, other clinical characteristics were similar in both groups and were not statistically significant. While the rate of prior nephrectomy before systemic treatment was 66.3% in the short-term group, this rate was 92.5% in the “long-term responders” group and difference was statistically significant (*P* ═ 0.006). Additionally, in comparison of short-term responders and long-term responders, number of patients with ECOG PS <1 was statistically significantly different as for 87.5% vs 51.8%, respectively (*P* values ═ 0.001). There were 25 patients with brain metastasis in the short-term responders’ group, while among the long-term responders’ group all patients had brain metastasis (*P* ═ 0.04).

### Survival outcomes and response rates

The overall population’s response rate (ORR) was 40.3%, and the disease control rate (DCR) was 75.3%; there was a statistically significant difference between the long and short-term responses (*P* ═ 0.001). Similarly, ORR and DCR were statistically significant in patients with favorable and intermediate-poor risk and with both short-term response and long-term response (*P* < 0.001) (see Table S3). Median PFS and OS for all patients were 12.5 months (95% CI, 8–11 months) and 76.4 months (95% CI, 49–104 months), respectively. As additional information, progression was observed in 257 patients after the first lines of treatment and 173 of all patients died during the follow-up.

**Figure 1.**
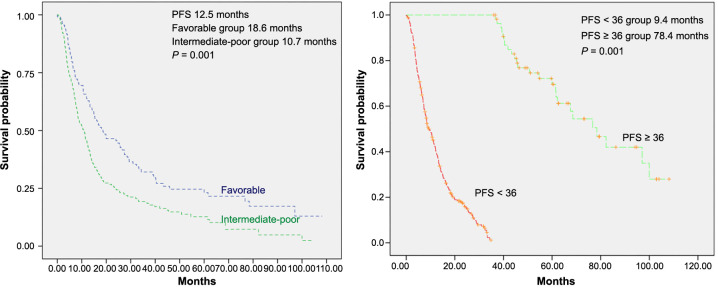
**Kaplan–Meier progression-free survival estimate according to IMDC score (A); according to TKI response (long-term vs short-term) (B); in all patients.** TKI: Tyrosine kinase inhibitor; IMDC: International Metastatic Renal Cell Carcinoma Database Consortium; PFS: Progression-free survival.

**Figure S2.**
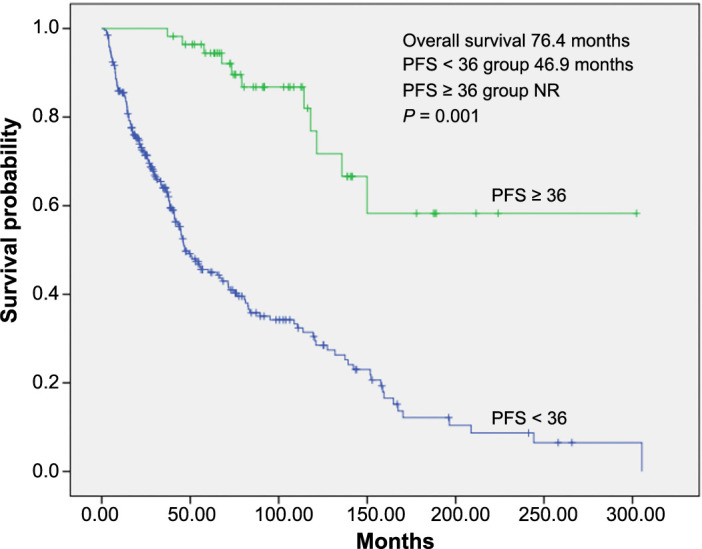
**Kaplan–Meier overall survival estimate according to tyrosine kinase inhibitor response (long-term vs short-term) in all patients.** PFS: Progression-free survival.

In long-term responders, the median PFS was 78.4 months (95% CI, 63–94 months), while in patients with PFS < 36 months, it was 9.4 months (95% CI, 36–58 months) (*P* ═ 0.001). The median PFS was 10.7 months (95% CI, 8–13 months) for the intermediate-poor risk population and 18.6 months (95% CI, 10–27 months) for the favorable risk group (Figure 1A and 1B). Median OS was not reached in long-term responders, whereas it was 46.9 months (95% CI, 36–58 months) in patients with PFS < 36 months (*P* ═ 0.001) (see Figure S2).

**Figure S3.**
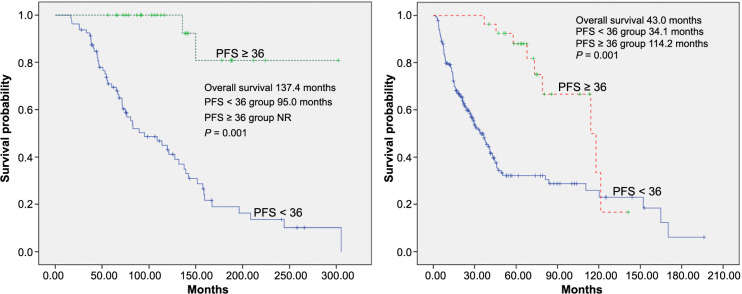
**Kaplan–Meier overall survival estimate according to tyrosine kinase inhibitor response (long-term vs short-term) in all patients in good risk (A) and intermediate-poor risk (B).** TKI: Tyrosine kinase inhibitor; PFS: Progression-free survival.

In the IMDC favorable risk group with PFS <36 months, the median OS was 95 months (95% CI, 57–133 months), whereas in the long-term responder group, it was non-reached (NR) (95% CI, NR) (*P* ═ 0.001). The median OS for intermediate-poor risk patients was 114 months (95% CI, 75–153 months) for long-term responders and 34 months (95% CI, 26–42 months) for short-term responders (see Figure S3). In addition, OS was 83 months (95% CI 51–113) in patients treated with sunitinib, while it was 67 months (95% CI 40–94) in the pazopanib arm (*P* ═ 0.19).

### Factors affecting long-term response

Three hundred twenty participants underwent logistic regression analysis to assess the relationships between clinical–pathological factors and long-term outcomes. Age, gender, histological type, prior nephrectomy, ECOG PS, sarcomatoid characteristics, IMDC score, and metastatic site were among the risk factors that were evaluated.

In univariate analysis, long-term responders were more likely to have had a previous nephrectomy, a better ECOG performance score, lower IMDC scores and be free of brain and bone metastases compared with short-term responders (*P* < 0.05, Table 2). In multivariate analysis, long-term responders were more likely to have had a previous nephrectomy, a better ECOG performance score, lower IMDC scores, receive less than 1 series of treatments, and absence of brain metastases compared with short-term responders (*P* < 0.05, Table 2).

**Table 2:** Univariate and multivariate analysis evaluating the relationship between long-term responders and clinicopathological factors

	**Univariate analysis**			**Multivariate analysis**		
	**HR**	**95% CI**	***P* value**	**HR**	**9** **5** **% CI**	***P*** **value**
Age > 70	0.83	0.36–1.88	0.66			
Gender, male	0.83	0.43–1.58	0.57			
Histology clear-cell RCC	0.79	0.58–1.06	0.12			
Previous nephrectomy, Yes	8.64	2.04–36.43	***0.003***	7.4	1.66–33.5	***0.009***
ECOG PS ≥ 1	0.52	0.29–0.93	***0.028***	0.51	0.26–0.98	0.04
Sarcomatoid feature, Yes	0.79	0.52–1.19	0.26			
IMDC score intermediate-poor	0.40	0.22–0.72	***0.002***			
*Metastatic sites,* *n* (%)						
Lung	0.97	0.53–1.75	0.92			
Liver	0.40	0.15–1.07	0.07			
Nodal	0.81	0.45–1.45	0.48			
Bone	0.43	0.22–0.82	***0.01***			
Brain	0.10	0.01–0.75	***0.02***	0.08	0.01–0.63	***0.01***
Line of therapy after TKI > 1, *n* (%)	0.11	0.05–0.21	***0.001***	0.26	0.13–0.5	***0.001***

ECOG PS: Eastern Cooperative Oncology Group performance status scale; RCC: Renal cell carcinoma; TKI: Tyrosine kinase inhibitor; IMDC: International Metastatic Renal Cell Carcinoma Database Consortium.

Univariate and multivariate analyses of the relationship between PFS ≥ 36 months and clinical–pathological variables in favorable and intermediate-poor risk patients are reported in Table S4 and Table 3. In multivariate analysis, the lack of brain metastases in long-term responders was statistically significant in the favourable risk group (OR, 0.12; 95% CI, 0.01–0.97; *P* ═ 0.04 (see Table S4). A significant differential effect of previous nephrectomy, not having received more than 1 series of treatment, and ECOG PS < 1 was observed in distinguishing intermediate-poor risk patients with and without PFS over 36 months both in univariate and multivariate analysis (*P* < 0.05, Table 3).

**Table 3:** Univariate and multivariate analysis evaluating the relationship between intermediate-poor risk long-term responders and clinicopathological factors

	**Univariate analysis**			**Multivariate analysis**		
	**HR**	**95% CI**	***P* value**	**HR**	**95% CI**	***P* value**
Age > 70	1.12	0.39–3.18	0.82			
Gender, male	0.45	0.19–1.05	0.06			
Histology clear-cell RCC	0.98	0.73–1.31	0.98			
Previous nephrectomy, Yes	6.35	1.4–27.6	**0** **.** **0** **1**	8.24	1.8–37.7	**0** **.** **0** **0** **7**
ECOG PS ≥ 1	0.40	0.16–0.94	**0** **.** **0** **3**	0.34	0.13–0.88	**0** **.** **0** **2** **7**
Sarcomatoid feature, Yes	0.72	0.4–1.32	0.29			
*Metastatic sites, n (%)*						
Lung	0.7	0.31–1.59	0.40			
Liver	0.66	0.21–2.04	0.48			
Nodal	1.1	0.49–2.48	0.80			
Bone	0.5	0.2–1.2	0.12			
Brain	0.01	0.00–1.1	0.90			
Line of therapy after TKI > 1, *n* (%)	0.21	0.08–0.53	0.11	0.13	0.05–0.35	**0** **.** **0** **0** **1**

ECOG PS: Eastern Cooperative Oncology Group Performance Status Scale; RCC: Renal cell carcinoma; TKI: Tyrosine kinase inhibitor.

## Discussion

In our study, we showed that patients with previous nephrectomy, ECOG PS < 1, and absence of brain metastases were treated with TKI alone to achieve a long-term response.

While TKIs were considered the standard of care in the treatment of metastatic RCC at the time when the patients participating in this study were treated [13, 14], today, combination treatments with immune checkpoint inhibitors have become the standard [5, 15–17]. The use of TKI monotherapy is suitable for limited cases [18, 19]. However, in the favorable risk population, combination therapies did not show a significant advantage in terms of OS over monotherapy TKI treatment, at the expense of greater toxicity [5, 15–17].

Although immunotherapy combinations are the standard first-line treatment for mRCC, most countries are unable to use them in first line for financial reasons [20]. And in real life, the usage rates of these combination regimens are very low. In developing countries like ours, combination therapies are not available for reimbursement. Therefore, in most of the world and in our country, TKIs are the standard treatment for first-line therapy in mRCC. Especially after the OS update analyses of the studies investigating the efficacy of combination therapies in patients in the favorable risk group showed that they did not contribute to survival, it is an important controversial issue in which patients in this group have short PFS and in which patient group in the intermediate and poor risk group long-term survival can be achieved [4, 21, 22]. Determining which patients in the intermediate and poor risk groups will benefit from single-agent TKIs is especially important for developing countries where access to immunoteropathics is difficult.

TKI therapy, which has been considered the standart in the first-line treatment of metastatic RCC for many years, is no longer considered a standart first-line treatment today. Its use as monotherapy for first-line treatment is still very limited.

In our study, we retrospectively examined the data of 320 patients diagnosed with metastatic RCC who received sunitinib and pazopanib in the first-line setting. Our aim was to investigate the clinicopathological characteristics of these patients, survival analyses, and factors affecting PFS and OS in long-term responders, as well as to conduct subgroup analyzes according to the IMDC risk score.

In the studies, the OS according to IMDC was 43.2 months, 22.5 months, and 7.8 months in the favorable group, intermediate group, and poor group, respectively [23]. In our study, median OS was found to be 76.4 months for the total population, 137 months for the IMDC favorable risk group, and 43 months for the IMDC intermediate-poor risk group, respectively.

Most of our patients were in the IMDC favorable and intermediate risk group (109 [34%] in IMDC 0, 85 [27%] in IMDC 1). In addition, 91 (28%) patients were treated with nivolumab in the second line. The OS duration of the patients was consistent with the literature and was found to be slightly longer. The reason for the long OS duration was that 82% of the patients were in the the favorable-intermediate group (IMDC 0 group-34%) and 28% of the patients used second-line immunotherapy.

However, although IMDC risk groups are currently the best prognostic factor, some of the favorable risk patients have a history of progression, while some of the patients in the intermediate-poor risk group have a very favorable prognosis. In particular, patients who have undergone nephrectomy, ECOG < 1 and do not have liver and brain metastases progress well [11, 12, 24]. Median OS was not reached in long-term responders. While the median OS could not be reached in the long-term responder with a favorable risk group, the median OS was calculated as 114 months in the long-term responder with an intermediate-poor risk group. The OS results in our study were longer than in other studies on this subject, which may reflect the existence of significant heterogeneity in the clinical-pathological characteristics of the patients [6, 24–28].

In our study, we found that nephrectomy, ECOG PS < 1, favorable risk, the absence of brain metastases, and no more than one series of treatment following TKI were all related with long-term responses. While the variables associated with long-term response in IMDC favorable risk patients included the absence of brain metastasis, in IMDC intermediate-poor risk patients, it was associated with nephrectomy, ECOG PS < 1, and not having received more than 1 series of treatment after TKI. In addition to the predictive risk factors determined by our study, laboratory parameters have been investigated in several recent studies. The outcomes differ depending on the IMDC risk factors [29, 30]

In our study, the general characteristics of patients with PFS ≥36 months with TKI treatment were similar to other studies [24, 31]. In one of these studies, the patient population with long-term response constituted 18.9% of all patients (in our study, this rate was 17.5%), and this group was the group that either received sunitinib treatment for more than 18 months or achieved a CR with sunitinib. The average duration of treatment with sunitinib was 24.9 months and the maximum duration was 73.9 months. In this study, long-term TKI response was associated with the absence of bone and lung metastases and being in the favorable risk group [24]. In another study, the rate of patients with long-term TKI response was found to be 19.3% and was associated with favorable risk patients <65 years of age with CR and PR [31].

By considering clinical–pathological variables associated with long-term responses, the best treatment decision can be made individually for each patient. The use of TKIs alone may still be safe, especially in favorable risk mRCC patients with low disease burden, slowly progressing disease, and no brain metastases. Although our study unfortunately has some limitations (such as the absence of a control group, being retrospective and some patient data not being accessible), we think that the study results should be taken into consideration due to its multicenter nature and high number of patients.

## Conclusion

In summary, TKIs can lead to longer survival in metastatic RCC patients. Predictors of long-term response, regardless of risk stratification, include prior nephrectomy, ECOG PS < 1, and absence of brain metastases. In the favorable risk group, the absence of brain metastases is a predictor of long-term response, while in the intermediate-poor risk group, prior nephrectomy and ECOG PS < 1 are predictors. Therefore, treatment decisions can be tailored based on each patient’s clinicopathological characteristics, and monotherapy with TKIs may be preferred as first-line treatment for some mRCC patient groups.

## Supplemental data

**Table S1:** Baseline characteristics of favorable risk patients according to TKI responses

	**All *n* ═ 109**	**PFS < 36 months *n* ═ 80 (73.4%) (short-term responder)**	***PFS* ≥ 36 months *n* ═ 29 (26.6%) (long-term responder)**	***P* value**
Age median (range)	59 (20–89)	60 (20–89)	53 (45–74)	0.41
Gender, *n* (%) male	82 (75.2)	58 (31.2)	24 (82.7)	0.24
Histology, *n* (%) clear cell RCC	89 (81.6)	61 (76.2)	28 (96.5)	0.32
Previous nephrectomy, *n* (%) Yes	107 (98.1)	78 (97.5)	29 (100)	0.39
ECOG PS, *n* (*%*) ≥ 1	49 (44.9)	38 (47.5)	11 (37.9)	0.34
Sarcomatoid feature Yes, *n* (%)	15 (13.7)	9 (11.2)	6 (20.6)	0.72
Metastatic sites, *n* (*%*)				
Lung	71 (65.1)	51 (63.7)	20 (68.9)	0.61
Liver	14 (12.8)	13 (16.2)	1 (3.4)	0.07
Nodal	48 (44.0)	38 (47.5)	10 (34.4)	0.22
Bone	31 (28.4)	24 (30.0)	7 (24.1)	0.62
Brain	16 (14.6)	15 (18.7)	1 (3.4)	***0.04***
*First-line therapy, n* (%)				
Sunitinib	78 (71.5)	61 (76.2)	17 (58.6)	0.07
Pazopanib	31 (28.4)	19 (23.7)	12 (41.3)	
Line of therapy after TKI, *n* (%) > 1	55 (50.4)	44 (55)	11 (37.9)	0.11

ECOG PS: Eastern Cooperative Oncology Group Performance Status scale; PFS: Progression-free survival; RCC: Renal cell carcinoma; TKI: Tyrosine kinase inhibitor.

**Table S2:** Baseline characteristics of intermediate-poor risk patients according to TKI responses

	**All *n* ═ 211**	**PFS < 36 months *n* ═ 184 (87.2%) (short-term responder)**	**PFS ≥ 36 months *n* ═ 27 (12.8%) (long-term responder)**	***P* value**
Age, median (range)	60 (29–83)	60 (29–83)	58 (31–81)	0.43
Gender, *n* (%) male	156 (73.9)	140 (76.0)	16 (59.2)	0.06
Histology, *n* (%)				
Clear cell RCC	176 (83.4)	153 (83.1)	23 (85.1)	0.71
Previous nephrectomy, *n* (%) Yes	147 (69.6)	122 (66.3)	25 (92.5)	***0.006***
ECOG PS, *n* (*%*) ≥ 1	175 (82.9)	161 (87.5)	14 (51.8)	**<** **0** **.** **0** **0** **1**
Sarcomatoid feature Yes, *n* (%)	31 (14.6)	28 (15.2)	3 (11.1)	0.22
*Metastatic sites, n (%)*				
Lung	125 (59.2)	111 (60.3)	14 (51.8)	0.41
Liver	42 (19.9)	38 (20.6)	4 (14.8)	0.47
Nodal	97 (45.9)	84 (45.6)	13 (48.1)	0.80
Bone	92 (43.6)	84 (45.6)	8 (29.6)	0.11
Brain	25 (11.8)	25 (13.5)	0	***0.04***
*First-line therapy, n (%)*				
Sunitinib	153 (72.5)	131 (71.1)	22 (81.4)	
Pazopanib	58 (27.4)	53 (28.8)	5 (18.5)	0.26
Line of therapy after TKI, *n* (%) > 1	121 (57.3)	114 (61.9)	7 (25.9)	**<** **0** **.** **0** **0** **1**

ECOG PS: Eastern Cooperative Oncology Group Performance Status scale; PFS: Progression-free survival; RCC: Renal cell carcinoma; TKI: Tyrosine kinase inhibitor.

**Table S3:** Treatment response rates of all patients, long and short-term responders

	**All patients *n* ═ 320**	**PFS < 36 months *n* ═ 264 (82.5%) (short-term responder)**	**PFS > 36 months *n* ═ 56 (17.5%) (long-term responder)**	***P* value**
Treatment response, *n* (%)				
Complete response	16 (5)	5 (1.8)	11 (19.6)	***<0.001***
Partial response	113 (35.3)	76 (28.7)	37 (66.0)	***<0.001***
Objective response rate	129 (40.3)	81 (30.6)	48 (85.7)	***<0.001***
Stable disease	112 (35)	104 (39.3)	8 (14.2)	***<0.001***
Disease control rate	241 (75.3)	185 (69.9)	56 (100)	***<0.001***

PFS: Progression-free survival.

**Table S4:** Univariate and multivariate analysis evaluating the relationship between favorable risk long-term responders and clinicopathological factors

	**Univariate analysis**			**Multivariate analysis**		
	**HR**	**95% CI**	***P* value**	**HR**	**95% CI**	***P* value**
Age > 70	0.59	0.15–2.25	0.44			
Gender, male	1.82	0.61–5.36	0.27			
Histology clear-cell RCC	0.26	0.03–1.78	0.17			
ECOG PS ≥ 1	0.67	0.28–1.61	0.37			
Previous nephrectomy, Yes	0.60	0.000–1.5	0.99			
Sarcomatoid feature, Yes	0.97	0.54–1.74	0.93			
*Metastatic sites, n (%)*						
Lung	1.26	0.5–3.13	0.61			
Liver	0.18	0.02–1.47	0.11			
Nodal	0.58	0.24–1.4	0.22			
Bone	0.41	0.15–1.12	0.08			
Brain	0.15	0.01–1.22	0.07	0.12	0.01–0.97	***0.04***
Line of therapy after TKI > 1, *n* (%)	0.5	0.21–1.19	0.11			

ECOG PS: Eastern Cooperative Oncology Group Performance Status scale; RCC: Renal cell carcinoma; TKI: Tyrosine kinase inhibitor.

## Data Availability

The datasets generated and/or analyzed during the current study are available from the corresponding author on reasonable request.
